# Longitudinal Changes in Neutrophil-to-Lymphocyte and Monocyte-to-Lymphocyte Ratios During Multiple Sclerosis Relapse

**DOI:** 10.3390/jcm15124539

**Published:** 2026-06-11

**Authors:** Marta Konieczna, Przemysław Puz, Michał Gałuszewski, Jan Olszewski, Karolina Jankowska, Alicja Gierlach, Krzysztof Wójcik, Anetta Lasek-Bal

**Affiliations:** 1 Department of Neurology, Upper Silesian Medical Center, Medical University of Silesia, 40-635 Katowice, Poland; 2 Department of Neurology, Faculty of Health Sciences in Katowice, Medical University of Silesia, Ziolowa 45, 40-635 Katowice, Poland; 3 Students’ Scientific Association, Department of Neurology, Faculty of Health Sciences in Katowice, Medical University of Silesia, 40-055 Katowice, Poland

**Keywords:** multiple sclerosis, relapse, neutrophil-to-lymphocyte ratio, monocyte-to-lymphocyte ratio

## Abstract

**Background:** The neutrophil-to-lymphocyte ratio (NLR) and monocyte-to-lymphocyte ratio (MLR) reflect the balance between innate and adaptive immunity and may indicate systemic inflammation. **Objectives:** To evaluate longitudinal changes in NLR and MLR in MS patients during relapse compared with relapse-free controls. **Methods:** In this retrospective single-center observational study, patients with MS were followed for 2 years. Individuals with at least one relapse and complete blood count data at predefined time points were included. Controls without relapse were selected in a 2:1 ratio and matched for age, sex, disease duration, EDSS score, and disease-modifying therapy category. NLR and MLR were assessed 3 months before relapse (or index date), during relapse, and 3 months after. **Results:** The study included 34 patients with relapse and 68 controls. During relapse, patients showed higher neutrophil and monocyte counts, lower lymphocyte counts, and elevated NLR and MLR compared with controls (all *p* < 0.001). NLR increased from 2.75 pre-relapse to 3.62 during relapse, then decreased to 2.67 (*p* = 0.0002). MLR rose from 0.45 to 0.54 and declined to 0.40 (*p* = 0.002). No significant changes were observed in controls. Conclusions: NLR and MLR demonstrated dynamic changes during MS relapse. These non-specific indices may serve as exploratory, complementary markers of relapse-associated inflammation, but require validation in larger prospective studies including MRI correlates.

## 1. Introduction

Multiple sclerosis (MS) is a chronic, progressive, inflammatory, demyelinating, and neurodegenerative disease of the central nervous system (CNS) [1,2]. Despite extensive research, the etiology of MS remains unclear; infectious, immunological, genetic, and environmental factors are all considered to contribute [3].

The pathogenesis of MS involves a complex interaction between the immune and nervous systems, in which increased pro-inflammatory activity leads to demyelination and neurodegeneration, ultimately resulting in disease relapses and disability progression [4].

The most common clinical phenotype, affecting approximately 85% of patients at diagnosis, is relapsing-remitting multiple sclerosis (RRMS) [1,5]. A relapse is clinically defined as the onset of new or worsening neurological symptoms lasting at least 24 h, occurring after a period of at least 30 days of clinical stability, and in the absence of infection or metabolic disturbance (pseudo-relapse) [1,3]. Relapses reflect disease activity, negatively affect quality of life, and contribute to disability progression (relapse-associated worsening, RAW).

A key element in relapse pathophysiology is transient disruption of the blood–brain barrier (BBB), allowing peripheral immune cells to infiltrate the CNS. Although MS has traditionally been viewed as a T- and B-cell–mediated disease, increasing evidence highlights the role of innate immune cells, including neutrophils and monocytes, in initiating the inflammatory cascade [6,7].

Interactions between innate and adaptive immune systems, glial cells, and neurons lead to inflammation, BBB disruption, demyelination, and neuroaxonal damage [6,7,8,9,10,11,12].

One of the main goals of disease-modifying therapy (DMT) in MS is to reduce relapse risk. Despite the availability of highly effective therapies (HET), complete elimination of relapses remains challenging, prompting the search for biomarkers associated with disease activity [13,14].

Recognized relapse triggers include infections, stress, and dysregulation of the hypothalamic–pituitary–adrenal axis [15,16,17,18]. Additional environmental factors such as smoking, air pollution (PM2.5), and vitamin D deficiency also contribute [19,20,21,22].

Identifying reliable biomarkers of disease activity remains a major challenge. Existing markers—including clinical measures, MRI findings, and laboratory parameters have limited sensitivity and specificity [23,24].

The neutrophil-to-lymphocyte ratio (NLR) and monocyte-to-lymphocyte ratio (MLR) are potential biomarkers reflecting the balance between innate and adaptive immunity [25,26,27,28,29]. Although previous studies suggest associations with relapse risk and disease progression, results remain inconsistent.

The primary aim of this study was to assess longitudinal within-patient changes in NLR and MLR before, during, and after relapse in patients with MS, compared with matched relapse-free controls assessed at corresponding index time points. A secondary exploratory aim was to evaluate whether baseline NLR and MLR measured before relapse were associated with subsequent relapse occurrence. This predictive analysis was considered hypothesis-generating because of the retrospective design and limited number of relapse cases.

Unlike most previous studies based on single time-point measurements, our study evaluates longitudinal changes in inflammatory indices at predefined intervals before, during, and after relapse, allowing assessment of their temporal dynamics in relation to clinical disease activity.

## 2. Materials and Methods

This was a retrospective, single-center observational study based on routinely collected clinical and laboratory data from patients with MS followed between 1 January 2024 and 31 December 2025.

The diagnosis of MS was established according to the 2017 McDonald criteria.

The study group included patients who experienced at least one relapse during the 24-month follow-up period and had complete blood count (CBC) results available at predefined time points. For patients with multiple relapses, only the first qualifying relapse with complete laboratory data was selected as the index relapse; therefore, each patient contributed only one set of measurements to the main analysis.

Exclusion criteria included: steroid therapy within 30 days prior to blood sampling; acute or chronic infection; malignancy; pregnancy; autoimmune, renal, hepatic, or thyroid disease; surgery within 3 months prior to sampling; hematological disorders; or blood transfusion within 3 months prior to sampling.

A relapse was defined as new or worsening neurological symptoms lasting ≥24 h, occurring in the absence of infection or fever, separated by at least 30 days from a previous relapse, and confirmed by a neurologist. The primary unit of analysis was the patient. Although some patients experienced more than one relapse during the 24-month follow-up period, only one relapse episode per patient was included in the main analysis. Therefore, each patient contributed only one set of measurements, corresponding to three time points: approximately 3 months before the index relapse, during the index relapse, and approximately 3 months after the index relapse.

The control group consisted of relapse-free patients selected from the same MS cohort. Controls were matched to relapse patients in a 2:1 ratio according to age, sex, disease duration, EDSS score, and DMT category. DMT category was classified as no treatment, low/moderate-efficacy therapy, or high-efficacy therapy. The matching procedure was used to improve comparability between groups and reduce confounding by major clinical and treatment-related variables.

For control patients, an index date corresponding to the matched relapse event was assigned to ensure comparable timing of laboratory assessments.

At each time point, infection was excluded based on clinical assessment, absence of fever or symptoms suggestive of acute infection, and normal CRP values at the time of blood sampling. Because of the retrospective design, no systematic microbiological screening was performed. Blood samples collected after initiation of corticosteroid therapy were excluded. In the relapse group, samples obtained during relapse were collected within 48 h of symptom onset and before steroid administration. All CBC measurements were performed in the same hospital laboratory using a standardized automated hematology analyzer (Sysmex NX 1000, Kobe, Japan) and routine laboratory procedures. The same laboratory method was used throughout the study period, and internal quality-control procedures were applied according to the laboratory’s standard operating protocols. Absolute lymphocyte, neutrophil, and monocyte counts were expressed as ×10^9^/L. NLR and MLR were calculated as dimensionless ratios by dividing the absolute neutrophil or monocyte count by the absolute lymphocyte count, respectively.

For each patient, three CBC measurements were analyzed.

In the relapse group:

- Time point 1: ~3 months (±7 days) before relapse;
- Time point 2: during relapse (≤48 h, before steroids);
- Time point 3: ~3 months (±7 days) after relapse.

In the control group:

- Time point 1: ~3 months before index date;
- Time point 2: index date;
- Time point 3: ~3 months after index date.

NLR and MLR were calculated at each time point. Data were verified for plausibility.

Relative changes were assessed using ratios: NLR2/NLR1, NLR3/NLR2, MLR2/MLR1, MLR3/MLR2.

Statistical analysis

Continuous variables are presented as mean ± standard deviation or median with range/interquartile range, depending on distribution and clinical convention. Baseline characteristics and between-group comparisons at individual time points were analyzed using Student’s *t*-test for normally distributed continuous variables or the Mann–Whitney U test for non-normally distributed variables. Categorical variables were compared using the chi-square test or Fisher’s exact test, as appropriate.

Longitudinal within-group changes in NLR and MLR across the three predefined repeated measurements were analyzed using the Friedman test. When the Friedman test was significant, post hoc pairwise comparisons between time points were performed using the Wilcoxon signed-rank test. Bonferroni correction was applied to these post hoc paired comparisons. Between-group differences in relative changes in NLR and MLR, expressed as NLR2/NLR1, NLR3/NLR2, MLR2/MLR1, and MLR3/MLR2, were analyzed using the Mann–Whitney U test.

Due to right-skewed distributions, NLR and MLR were log-transformed where parametric modeling was applied. A two-sided *p*-value < 0.05 was considered statistically significant, except for Bonferroni-adjusted post hoc comparisons, where *p* < 0.0167 was used.

To address the potential confounding effect of disease-modifying therapies (DMTs), additional analyses were performed. First, a sensitivity analysis was conducted excluding patients treated with therapies known to significantly affect lymphocyte counts (dimethyl fumarate, sphingosine-1-phosphate receptor modulators, anti-CD20 therapies, and cladribine). All primary analyses were repeated in this restricted cohort. Second, patients in the relapse group were stratified according to DMT mechanism of action into two groups: (1) lymphocyte-altering therapies and (2) non–lymphocyte-altering therapies. Changes in NLR and MLR between time points were compared between these groups. Exploratory logistic regression analyses were performed to assess whether baseline inflammatory markers measured approximately three months before relapse were associated with subsequent relapse occurrence. Because of the limited number of relapse cases, the models were deliberately restricted to reduce the risk of overfitting. Clinical covariates included age, sex, disease duration, EDSS and highly effective treatment. Inflammatory markers were entered separately into the models to avoid collinearity between individual leukocyte counts and derived ratios. Separate models were therefore fitted for NLR1 and MLR1. Results are presented as odds ratios with 95% confidence intervals. These analyses were considered exploratory.

Statistical analyses were performed using Statistica version 13.0 (TIBCO Software Inc., Palo Alto, CA, USA).

## 3. Results

### 3.1. Patient Characteristics

During the study period, 512 patients were treated in the center; 38 patients experienced at least one relapse. After applying the inclusion and exclusion criteria, 34 patients with relapse and 68 relapse-free matched controls were included in the analysis. The 34 patients in the relapse group experienced a total of 53 relapses during the observation period; however, in patients with multiple relapses, the first qualifying relapse with complete laboratory data at all predefined time points was selected. Accordingly, the relapse group is presented as *n* = 34 in all tables. After 2:1 matching, the relapse and control groups were comparable with respect to age, sex distribution, disease duration, EDSS score, and DMT category, as shown in Table 1.

### 3.2. Between-Group Differences in Hematological Parameters at Each Time Point

Between-group comparisons of absolute hematological parameters at each predefined time point are shown in Table 2. At baseline, approximately three months before relapse or index date, NLR and MLR did not differ significantly between patients with relapse and relapse-free controls. During the relapse period, patients experiencing relapse had significantly lower lymphocyte counts and significantly higher neutrophil counts, monocyte counts, NLR, and MLR compared with relapse-free controls at the corresponding index time point. (Table 2). At the follow-up time point, approximately three months after relapse or index date, no significant between-group differences in NLR or MLR were observed.

These findings suggest that dynamic changes in NLR and MLR may be associated with clinical relapse activity in this cohort, although the modest sample size limits definitive interpretation.

### 3.3. Within-Group Longitudinal Changes in NLR and MLR

**Figure 1:**
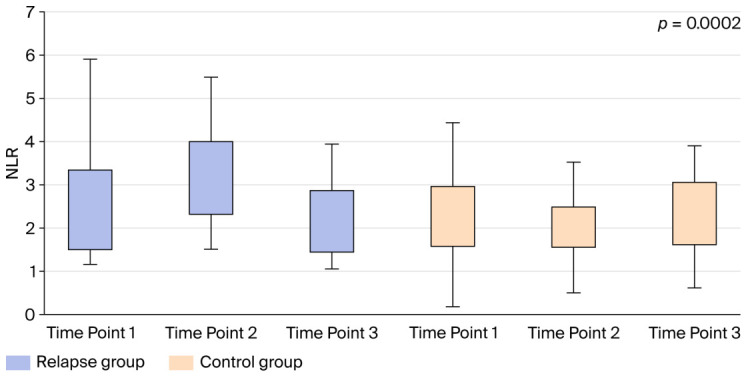
Longitudinal changes in NLR values across three predefined time points in patients with relapse and relapse-free controls. Time point 1: approximately 3 months before relapse/index date; Time point 2: relapse/index date; Time point 3: approximately 3 months after relapse/index date.

In the relapse group, significant changes in NLR and MLR values were observed in subsequent measurements; during the relapse period, a statistically significant increase in NLR and MLR was observed compared to measurements taken 3 months prior, and during the 3 months following relapse, a decrease in NLR and MLR values was observed compared to the relapse period. 3 months before the relapse, the mean NLR was 2.75; during the relapse, it was 3.62; and 3 months after the relapse, it was 2.67 (*p* = 0.0002, Figure 1).

**Figure 2:**
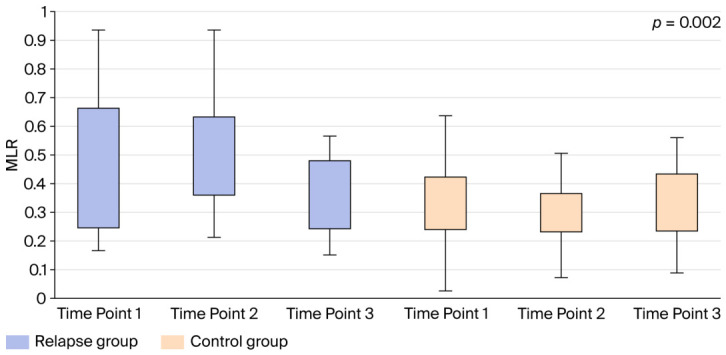
Longitudinal changes in MLR values across three predefined time points in patients with relapse and relapse-free controls. Time point 1: approximately 3 months before relapse/index date; Time point 2: relapse/index date; Time point 3: approximately 3 months after relapse/index date.

The mean MLR was 0.45 three months before relapse, 0.54 at the time of relapse, and 0.4 three months after relapse (*p* = 0.002, Figure 2).

Post hoc paired comparisons confirmed a significant increase in NLR and MLR from the pre-relapse period to relapse and a significant decrease from relapse to the post-relapse period (*p* < 0.001).

No statistically significant changes in NLR and MLR were observed in the measurements repeated every 3 months in patients without relapse (Figure 1 and Figure 2).

A comparative analysis of NLR and MLR indices during the relapse period, as compared to measurements taken 3 months before and after relapse, revealed significant differences between the relapse group and the control group (Table 3).

Relative-change ratios were calculated as the value at the later time point divided by the value at the preceding time point. NLR2/NLR1 and MLR2/MLR1 represent changes from baseline to relapse/index date. NLR3/NLR2 and MLR3/MLR2 represent changes from the relapse/index date to follow-up. NLR = neutrophil-to-lymphocyte ratio; MLR = monocyte-to-lymphocyte ratio.

In the sensitivity analysis excluding patients treated with lymphocyte-altering therapies, the overall pattern of findings was similar to that observed in the main analysis (Table S1). Patients experiencing relapse continued to show higher NLR and MLR values during relapse compared with controls. Similarly, within the relapse group, NLR and MLR values increased significantly during relapse compared to pre-relapse measurements and decreased during follow-up.

In the stratified analysis according to DMT mechanism of action, both groups (lymphocyte-altering vs. non–lymphocyte-altering therapies) showed a comparable pattern of increase in NLR and MLR during relapse (Table S2). No statistically significant differences were observed between groups in the magnitude of these changes, as assessed by relative changes ratios.

As a secondary exploratory analysis, we assessed whether baseline inflammatory markers measured approximately three months before relapse were associated with subsequent relapse occurrence. Neither baseline NLR nor baseline MLR was independently associated with relapse occurrence after adjustment for age, sex, and highly effective treatment. Therefore, baseline NLR and MLR did not demonstrate independent short-term predictive value in this cohort (Tables S3 and S4). Given the exploratory nature of the regression analysis and the limited number of relapse cases, we did not interpret the AUC as evidence of clinically useful predictive performance. The analysis did not identify baseline NLR or MLR as independent short-term predictors of relapse.

## 4. Discussion

The main findings of our study are the presence of significant changes in the cellular composition of peripheral blood during MS relapse and the demonstration of dynamic changes in NLR and MLR compared with periods of remission.

In the relapse group, lymphocyte counts were significantly lower, whereas neutrophil and monocyte counts were significantly higher compared with the index time point in appropriately matched patients without relapse. These differences were not observed in measurements performed three months before relapse or three months after its resolution, indicating a close association with current disease activity. Because the main analysis was conducted at the patient level and included only one index relapse per patient, multiple relapse episodes from the same individual were not treated as independent observations. This approach avoided inflation of the sample size and eliminated the need to account for within-patient correlation between multiple relapse events.

The use of NLR and MLR as inflammatory markers may be more clinically informative than the assessment of individual leukocyte populations alone, as these ratios reflect the balance between innate and adaptive immune responses. This approach reduces the influence of isolated fluctuations in leukocyte subpopulations that may occur due to non-specific factors.

Previous studies have demonstrated higher NLR and MLR values in patients with MS compared with healthy controls [9,30,31]. In the study by Fahmi et al., higher NLR values were observed both in MS patients compared with healthy individuals and in progressive MS compared with RRMS. Additionally, a positive correlation between NLR and disability (EDSS) was reported [30].

The increase in NLR observed during relapse in our cohort is consistent with findings from other studies [8,31,32]. In a large study of 641 patients, higher baseline NLR and MLR values were associated with an increased risk of relapse over a two-year follow-up period [25]. In contrast, Yetkin et al. did not find a direct association between NLR and relapse risk but demonstrated that higher NLR values were associated with a more aggressive disease course, as reflected by the need for treatment escalation [33].

An association between elevated NLR and MLR values and disability progression has also been reported [27,29]. In the study by Hemond et al., including patients with different MS phenotypes and various DMTs, both ratios were associated with progressive disease, reduced quality of life, and increased brain atrophy on MRI [27]. Conversely, Bisgaard et al. did not confirm a relationship between NLR and EDSS or MS phenotype but did demonstrate higher NLR values in MS patients compared with healthy controls, supporting its role as a marker of systemic inflammation [34]. The discriminatory significance of elevated NLR values for the onset and activity of MS was also confirmed in experimental studies using models of experimental autoimmune encephalomyelitis [35].

Discrepancies between studies regarding the predictive value of NLR and MLR likely result from differences in study design, patient populations, disease phenotypes, DMT exposure, timing of laboratory measurements, and sample size. Certain treatments, particularly anti-CD20 therapies and sphingosine-1-phosphate receptor modulators, may significantly alter lymphocyte and leukocyte profiles. Although groups were matched for DMT category, residual confounding cannot be excluded. Therefore, the observed changes in NLR and MLR should be interpreted with caution, and future studies should include treatment-stratified analyses.

An important methodological consideration in the interpretation of our findings is the potential confounding effect of disease-modifying therapies, particularly those with mechanisms directly influencing lymphocyte trafficking or depletion. Agents such as sphingosine-1-phosphate receptor modulators, anti-CD20 therapies, and cladribine may substantially alter peripheral blood cell counts, thereby potentially affecting derived inflammatory indices such as NLR and MLR. To address this issue, we performed additional analyses excluding patients treated with lymphocyte-altering therapies, as well as stratified analyses based on DMT mechanism of action. Importantly, the observed increase in NLR and MLR during relapse remained consistent after exclusion of these therapies and did not differ significantly between treatment groups.

These findings suggest that the observed dynamic changes in NLR and MLR are not explained exclusively by DMT-related alterations in leukocyte profiles. However, DMT-related confounding cannot be fully excluded, particularly given the heterogeneity of treatment mechanisms and the limited number of patients in treatment-defined subgroups. Therefore, the sensitivity and stratified analyses should be considered exploratory and supportive rather than conclusive.

Importantly, most previously published studies have relied on single baseline measurements of NLR and MLR. In contrast, our study included repeated measurements at defined time points, allowing assessment of their temporal dynamics. This approach provides a more comprehensive understanding of the behavior of these markers over time.

An important finding of our study is the lack of predictive value of baseline NLR and MLR measured three months prior to relapse. This suggests that these indices are not suitable as standalone prognostic biomarkers for short-term relapse risk. Instead, their value appears to lie in reflecting ongoing inflammatory activity. This distinction is clinically relevant, as it indicates that NLR and MLR should be interpreted as dynamic markers of current disease state rather than predictors of future events.

Our findings support the hypothesis that NLR and MLR may behave as dynamic inflammatory indices during MS relapse. A significant increase was observed during relapse compared with both pre-relapse and post-relapse periods. In contrast, no significant fluctuations were observed in the control group, supporting the stability of these indices in patients without active disease.

Similar observations were reported by Nikseresht and Bahrami, who demonstrated increased NLR during relapse and a decrease during follow-up [36].

The analysis of relative changes (e.g., NLR2/NLR1 and MLR2/MLR1) further emphasizes the importance of monitoring trends over time rather than relying on absolute values. These dynamic changes may better reflect underlying inflammatory processes associated with relapse.

From a pathophysiological perspective, elevated NLR and MLR during relapse reflect activation of innate immune mechanisms. Neutrophils contribute to MS pathogenesis through early CNS infiltration, increased BBB permeability, production of reactive oxygen species and proteolytic enzymes, formation of neutrophil extracellular traps, and amplification of inflammatory responses, including promotion of Th17 pathways [37]. Monocytes also play a key role by migrating across the BBB, differentiating into macrophages, producing pro-inflammatory cytokines and reactive oxygen species, and presenting antigens to T lymphocytes, thereby sustaining CNS inflammation [37,38,39].

From a clinical perspective, the applicability of NLR and MLR lies primarily in their availability and low cost, as they are derived from routine blood tests. However, their role should be considered complementary rather than substitutive to established measures such as MRI and clinical assessment. Further studies are needed to determine whether integrating these indices into clinical algorithms improves decision-making. An important limitation of the present study is the absence of systematically collected MRI activity data at the predefined time points. Therefore, we could not assess whether the observed changes in NLR and MLR were associated with gadolinium-enhancing lesions or new/enlarging T2 lesions. Consequently, our findings should be interpreted as reflecting hematological changes associated with clinically defined relapse rather than radiological disease activity or MS disease activity more broadly.

In our study, baseline hematological parameters measured three months prior to relapse did not predict relapse occurrence. This suggests that these indices may better reflect ongoing relapse-associated inflammatory activity than predict short-term relapse risk. However, because the regression analysis was exploratory and limited by the small number of relapse cases, larger prospective studies are needed before definitive conclusions regarding predictive utility can be drawn.

The strengths of this study include the use of real-world clinical data, a two-year observation period, and regular, standardized blood sampling every three months. The longitudinal design allowed for assessment of dynamic changes in inflammatory markers, which distinguishes this study from previous analyses based on single measurements.

Limitations

The main limitations of this study are its retrospective, single-center design and relatively small sample size, particularly in subgroup and sensitivity analyses; therefore, the results should be interpreted as exploratory and hypothesis-generating. Despite matching for major clinical variables and DMT category, residual confounding cannot be excluded. MRI activity was not systematically assessed, so the relationship between NLR/MLR dynamics and radiological disease activity could not be evaluated. Although some patients had more than one relapse, the primary analysis was performed at the patient level using one index relapse per patient, which avoided non-independence of observations but precluded analysis of recurrent events. Data on other potential confounders, including smoking, BMI, hormonal factors, subclinical infections, and cytokine profiles, were unavailable. The small number of patients receiving individual DMTs prevented treatment-specific subgroup analyses, although DMT distribution, including therapies affecting blood counts, did not differ between groups. The exploratory regression analyses were also limited by the small number of relapse cases and were underpowered to develop or validate a robust prediction model. Larger prospective studies are needed to confirm whether relapse-associated NLR and MLR changes are independent of treatment-related effects and whether they correlate with MRI activity.

Overall, NLR and MLR may reflect relapse-associated systemic inflammation, but they should be regarded as exploratory markers complementary to clinical assessment and MRI, not as standalone indicators of MS activity.

## 5. Conclusions

In this retrospective single-center cohort, NLR and MLR showed significant longitudinal changes during MS relapse, with increases during relapse and decreases during follow-up. These findings suggest that NLR and MLR may reflect relapse-associated systemic inflammatory activity. However, because these indices are non-specific and the sample size was modest, they should be interpreted as exploratory and hypothesis-generating rather than established biomarkers. Larger prospective studies incorporating MRI activity, standardized sampling, and treatment-stratified analyses are needed to validate their clinical utility.

## Figures and Tables

**Table 1:** Characteristics of patients with a relapse and the control group.

Characteristics	Patients with Relapse *n* = 34	Control Group *n* = 68	*p*
Age	41.97 ± 12.23	43.91 ± 10.91	0.43
Sex (Female), *n*(%)	20/34 (58.8%)	49/68 (72.1%)	0.18
EDSS [median, min–max]	2 [1,2,3,4,5]	2 [0–5.5]	0.78
Disease duration	5.39 ± 4.33	5.21 ± 3.42	0.77
Disease modifying treatment	none: 3 interferons -3 glatiramer acetate -3 dimethyl fumarate -16 teriflunomide -4 ocrelizumab -1 cladribine -2 S1P modulators -2	interferons -13 glatiramer acetate -5 dimethyl fumarate -25 teriflunomide -10 ofatumumab -4 ocrelizumab -3 natalizumab -3 S1P modulators -4 cladribine -1	0.62

EDSS—Expanded Disability Status Scale, S1P-sphingosine-1-phosphate.

**Table 2:** Inflammatory markers in patients experiencing a relapse and in the control group.

Parameter	Patients with Relapse *n* = 34	Control Group *n* = 68	*p*
Lymphocyte count 1	1.41 ± 0.52	1.68 ± 0.66	0.07
Neutrophil count 1	3.45 ± 1.26	3.50 ± 1.24	0.83
Monocyte count 1	0.57 ± 0.27	0.56 ± 0.23	0.95
NLR 1	2.75 ± 1.3	2.42 ± 1.44	0.19
MLR 1	0.45 ± 0.23	0.38 ± 0.26	0.13
Lymphocyte count 2	1.41 ± 0.49	1.79 ± 0.58	<0.001
Neutrophil count 2	4.52 ± 1.44	3.34 ± 1.03	<0.001
Monocyte count 2	0.69 ± 0.26	0.54 ± 0.18	<0.001
NLR 2	3.62 ± 1.92	1.99 ± 0.67	<0.001
MLR 2	0.54 ± 0.27	0.32 ± 0.16	<0.001
Lymphocyte count 3	1.57 ± 0.75	1.69 ± 0.77	0.44
Neutrophil count 3	3.11 ± 0.91	3.56 ± 1.38	0.22
Monocyte count 3	0.51 ± 0.15	0.55 ± 0.22	0.50
NLR 3	2.67 ± 2.72	2.34 ± 0.99	0.43
MLR 3	0.40 ± 0.24	0.37 ± 0.21	0.71

NLR—Neutrophil-to-Lymphocyte Ratio; MLR—Monocyte-to-Lymphocyte Ratio.

**Table 3:** Relative changes in NLR and MLR between consecutive time points.

Parameter	Patients with Relapse *n* = 34	Control Group *n* = 68	*p*
NLR2/NLR1	1.31 ± 0.74	1.04 ± 1.15	0.049
MLR2/MLR1	1.7 ± 1.12	0.97 ± 0.31	<0.001
NLR3/NLR2	0.83 ± 1.17	1.27 ± 0.86	<0.001
MLR3/MLR2	0.71 ± 0.09	1.12 ± 0.23	<0.001

## Data Availability

The data presented in this study are not publicly available due to privacy restrictions.

## Supplementary Materials

The following supporting information can be downloaded at: https://www.mdpi.com/article/10.3390/jcm15124539/s1, Table S1: Sensitivity analysis after exclusion of patients treated with lymphocyte-altering therapies; Table S2: Changes in NLR and MLR according to DMT mechanism of action. Table S3: Logistic regression analysis: baseline NLR and clinical data as predictive factors for relapse during the 3-month observation period; Table S4: Logistic regression analysis: baseline MLR and clinical data as predictive factors for relapse during the 3-month observation period.

## Abbreviations

The following abbreviations are used in this manuscript:

NLR	neutrophil-to-lymphocyte ratio
MLR	monocyte-to-lymphocyte ratio
MS	multiple sclerosis
CNS	central nervous system
RRMS	relapsing-remitting multiple sclerosis
RAW	relapse-associated worsening
BBB	blood–brain barrier
DMT	disease-modifying therapy
HET	highly effective therapies
CBC	complete blood count
