# Supplementary material for: Longitudinal Changes in Neutrophil-to-Lymphocyte and Monocyte-to-Lymphocyte Ratios During Multiple Sclerosis Relapse
Source: J Clin Med. 2026 Jun 11;15(12):4539. doi: 10.3390/jcm15124539 (PMC13302109; doi:10.3390/jcm15124539)
Supplement: Supplementary file 1 [file jcm-15-04539-s001.zip › jcm-4314506-supplementary.pdf]

Table S1. Sensitivity analysis after exclusion of patients treated with lymphocyte-altering therapies

Parameter	Patients with relapse n=13	Control group n=31	p
NLR 1	2.32±1.16	2.17±1.19	0.77
MLR 1	0.35±0.21	0.36±0.21	0.72
NLR 2	2.97±0.92	1.87±0.73	<0.001
MLR 2	0.45±0.14	0.32±0.1	<0.001
NLR 3	3.46±4.39	2.27±1.02	0.78
MLR 3	0.43±0.28	0.37±0.18	0.60

NLR = Neutrophil-to-Lymphocyte Ratio; MLR = monocyte-to-lymphocyte ratio

Table S2. Changes in NLR and MLR according to DMT mechanism of action.

Parameter	Lymphocyte-altering therapies n=21	Non–lymphocyte-altering therapies n=13	p
NLR2/NLR1	1.23±0.84	1.13±0.47	0.76
MLR2/MLR1	1.97±1.18	1.45±0.67	0.28
NLR3/NLR2	0.67±0.3	1.29±1.99	0.8
MLR3/MLR2	0.77±0.05	1.02±0.13	0.58

NLR = neutrophil-to-lymphocyte ratio; MLR = monocyte-to-lymphocyte ratio; DMT = disease-modifying therapy

Table S3. Logistic regression analysis: baseline NLR and clinical data as predictive factors for relapse during the 3-month observation period.

Predicting factor	OR	95% CI	p
Age (for each year)	1.08	0.77-3.14	0.92
Female sex	1.11	0.85-1.78	0.55
HET	0.81	0.55-1.05	0.11
Disease duration (each year)	0.9	0.76-10.07	0.37
EDSS/ each point	1.17	0.73-2.97	0.52
NLR 1	1.12	0.42-4.13	0.46

Table S4. Logistic regression analysis: baseline MLR and clinical data as predictive factors for relapse during the 3-month observation period.

Predicting factor	OR	95% CI	p
Age (for each year)	1.14	0.56-2.01	0.77
Female sex	1.06	0.7-1.38	0.51
HET	0.21	0.12-3.43	0.16
Disease duration (each year)	1.03	0.67-3.72	0.56
EDSS/ each point	1.05	0.61-1.86	0.51
MLR 1	1.21	0.45-3.94	0.89
